# Identification of Novel Molecular Markers of Human Th17 Cells

**DOI:** 10.3390/cells9071611

**Published:** 2020-07-03

**Authors:** Anna Sałkowska, Kaja Karaś, Iwona Karwaciak, Aurelia Walczak-Drzewiecka, Mariusz Krawczyk, Marta Sobalska-Kwapis, Jarosław Dastych, Marcin Ratajewski

**Affiliations:** 1 Laboratory of Epigenetics, Institute of Medical Biology, Polish Academy of Sciences, 93-232 Lodz, Poland; asalkowska@cbm.pan.pl (A.S.); kaja.karas@gmail.com (K.K.); 2 Laboratory of Transcriptional Regulation, Institute of Medical Biology, Polish Academy of Sciences, 93-232 Lodz, Poland; isachrajda@cbm.pan.pl; 3 Laboratory of Cellular Immunology, Institute of Medical Biology, Polish Academy of Sciences, 93-232 Lodz, Poland; adrzewiecka@cbm.pan.pl (A.W.-D.); jdastych@cbm.pan.pl (J.D.); 4 Genomed S.A., 12 Ponczowa Street, 02-971 Warsaw, Poland; mkraw@genomed.pl; 5 Biobank Lab, Department of Molecular Biophysics, Faculty of Biology and Environmental Protection, University of Lodz, 90-237 Lodz, Poland; marta.sobalska@biol.uni.lodz.pl; 6 BBMRI.pl Consortium, 54-066 Wroclaw, Poland

**Keywords:** Th17 cells, *APOD*, *C1QL1*, *CTSL*

## Abstract

Th17 cells are important players in host defense against pathogens such as *Staphylococcus aureus*, *Candida albicans*, and *Bacillus anthracis*. Th17 cell-mediated inflammation, under certain conditions in which balance in the immune system is disrupted, is the underlying pathogenic mechanism of certain autoimmune disorders, e.g., rheumatoid arthritis, Graves’ disease, multiple sclerosis, and psoriasis. In the present study, using transcriptomic profiling, we selected genes and analyzed the expression of these genes to find potential novel markers of Th17 lymphocytes. We found that *APOD* (apolipoprotein D); *C1QL1* (complement component 1, Q subcomponent-like protein 1); and *CTSL* (cathepsin L) are expressed at significantly higher mRNA and protein levels in Th17 cells than in the Th1, Th2, and Treg subtypes. Interestingly, these genes and the proteins they encode are well associated with the function of Th17 cells, as these cells produce inflammation, which is linked with atherosclerosis and angiogenesis. Furthermore, we found that high expression of these genes in Th17 cells is associated with the acetylation of H2BK12 within their promoters. Thus, our results provide new information regarding this cell type. Based on these results, we also hope to better identify pathological conditions of clinical significance caused by Th17 cells.

## 1. Introduction

Th17 cells are one of three separate subsets of CD4+ effector lymphocytes (the other two are Th1 and Th2 cells). Th17 cells are characterized by the cytokines they produce (IL17A, IL17F, IL21, IL22) [1,2,3,4], their transcription factors (RORγT) [5,6], and their receptors (CD161, CCR4, CCR6) [7,8]. Th17 cells mediate protective adaptive immunity against *Bacillus anthracis* [9], *Staphylococcus aureus* [10], and *Candida albicans* [11]. RORγT, considered the master transcription factor, orchestrates the differentiation of Th17 lymphocytes and regulates expression of the signature cytokine interleukin 17 [6]. The expression of RORγT is dependent on STAT3 [12], and perturbations in STAT3 signaling affect the development of Th17 lymphocytes. A good example of the importance of STAT3 can be seen in the differentiation of Th17 cells observed in patients suffering from Job’s syndrome (also known as hyperimmunoglobulin E syndrome (HIES)), which is a primary immune deficiency disorder characterized by chronic mucocutaneous candidiasis and recurring pneumonia caused by *Candida albicans* and *Staphylococcus aureus*, respectively [13]. This deficiency is the effect of mutations within the *STAT3* gene [14,15] that lead to the very low expression of RORγT and the absence of Th17 cells [16]. However, despite their important physiological role in humans, Th17 cells are known mainly for their negative role over the course of numerous autoimmune diseases, including rheumatoid arthritis [17], multiple sclerosis [18], psoriasis [19], inflammatory bowel disease [20], Graves’ disease [21], ankylosing spondylitis [22], and Crohn’s disease [23].

Some known Th17 markers are also expressed by other cells of the immune system because their expression is not completely restricted to Th17 cells or because of phenotypic and functional plasticity (the transition of one cell type to another) [24,25,26,27]. The aim of the present study was to find new markers of Th17 cells that could be of clinical relevance to identify inflammation caused by these lymphocytes. Using a transcriptomic approach, we selected several candidate genes, the expression of which at the mRNA and protein levels was then analyzed in Th1, Th2, Th17, and Treg cells. The results of this analysis indicated that *APOD* (apolipoprotein D); *C1QL1* (complement component 1, Q subcomponent-like protein 1); and *CTSL* (cathepsin L) show Th17-specific expression. Furthermore, the products of *APOD*, *C1QL1*, and *CTSL* are secreted proteins, suggesting their potential usefulness for monitoring Th17 cell-driven inflammation in a clinical setting.

## 2. Materials and Methods

### 2.1. Naive CD4+ TCell Isolation and Differentiation

Peripheral blood mononuclear cells were isolated from buffy coats obtained from healthy, anonymous donors by Ficoll gradient centrifugation. The naive CD4+ fraction was isolated using CD4 M-pluriBead^®^ anti-Hu beads (pluriSelect Life Science, Leipzig, Germany). Human Th1 cells were generated using the Human Th1 Cell Differentiation Kit (R&D Systems, CDK001, Minneapolis, MN, USA) and then maintained in RPMI 1640 medium supplemented with 5% FBS, 2 mM L-glutamine, 50 units/mL penicillin, 50 µg/mL streptomycin, 50 µM 2-ME with Human Th1 Reagent 1 and Human Th1 Reagent 2 (part of the Human Th1 Cell Differentiation Kit) for 5 days. Human Th2 cells were generated using the Human Th2 Cell Differentiation Kit (R&D Systems, CDK002) and then maintained in RPMI 1640 medium supplemented with 100 units/mL penicillin and 100 µg/mL streptomycin with Human Th2 Reagents 1, 2, 3 and 4 (part of the Human Th2 Cell Differentiation Kit) for 13 days. Th17 cells were obtained according to the protocol described by Wilson et al. [28] and cultured in Yssel’s medium containing human AB serum, anti-CD2/anti-CD3/anti-CD28 beads (T cell activation/expansion kit from MiltenyiBiotec) and the cytokines human IL-1b (50 ng/mL), human IL-6 (30 ng/mL), human IL-23 (10 ng/mL), and human transforming growth factor β (TGF-β) (10 ng/mL) for 5 days. To isolate Tregs, the CD4+CD25+CD127dim/- Regulatory T Cell Isolation Kit II (MiltenyiBiotec) was used. Cells were then cultured in Yssel’sTcell medium with 1% human serum AB supplemented with 2 ng/mL TGFB, 5 ng/mL IL-2, and beads (Treg Expansion Kit from MiltenyiBiotec) for 14 days. The cytokines used in the present study were purchased from PeproTech (Rocky Hill, NJ, USA).

### 2.2. RNA Sequencing (RNA-seq) and Analysis of Differentially Expressed Genes (DEGs)

Global changes in gene expression in human naive CD4+ cells and fully differentiated Th17 cells (from three anonymous blood donors) were analyzed by high-resolution RNA sequencing (RNA-seq). For each sample, the mRNA fraction was isolated with a NEBNext^®^ Poly(A) mRNA Magnetic Isolation Module Kit (New England Biolabs, Ipswich, MA, USA) according to the manufacturer’s instructions. Libraries were prepared using the NEBNext^®^ Ultra™ RNA Library Prep Kit for Illumina^®^ (New England Biolabs) according to the manufacturer’s instructions. Sequencing was performed on a HiSeq2000 instrument (Illumina, San Diego, CA, USA) in PE100 mode. FASTQ sequence reads were aligned to the GRCh38 reference genome. Adapter trimming was performed using the bbduk script (https://sourceforge.net/projects/bbmap/). Prior to DEG analysis, the gene expression statistics were analyzed using Salmon software [29], which provides fast and bias-aware quantification of transcript expression. The quantification of gene expression was performed at the transcript level. Then, quantified transcript-level data from the two datasets (CD4+ cells vs Th17 cells) were aggregated at the gene level for gene-level differential expression and analyzed using the R package DESeq. (https://bioconductor.org/packages/release/bioc/html/DESeq.html; [30]). Benjamini and Hochberg’s approach was used to control the false discovery rate and adjust the *p*-values. An adjusted *p*-value < 0.05 and fold change in expression of 1.5 were the criteria used to define significant differences in gene expression.

### 2.3. Gene Ontology

Gene ontology analysis was performed using PANTHER software [31].

### 2.4. Quantitative PCR

RNA was isolated from cells using TRI Reagent (Molecular Research Center, Cincinnati, OH, USA). Five micrograms of RNA was used as a template for reverse transcription reactions performed using the Maxima First Strand cDNA Synthesis Kit for RT-qPCR (Thermo Fisher Scientific, Waltham, MA, USA). Real-time RT-PCR was performed using SYBR Green I Master Mix on a LightCycler 480 from Roche (Basel, Switzerland). The reactions were run in a 384-well white plate at 95 °C for 5 min, followed by 45 cycles of 95 °C for 10 s, 60 °C for 10 s, and 72 °C for 20 s. The following primers pairs were used: RORγT, 5′-CTGCTGAGAAGGACAGGGAG-3′ (forward) and 5′-AGTTCTGCTGACGGGTGC-3′ (reverse), described previously [32]; GATA3, 5′-TGTCTGCAGCCAGGAGAGCAG-3′ (forward) and 5′-TGGTGTGGTCCAAAGGACAGG-3′ (reverse), described previously [33]; TBX21, 5′-AGCTCACAAACAACAAGGGG-3′ (forward) and 5′-ATTCTGGTAGGCAGTCACGG-’3 (reverse); FOXP3, 5′-AGGGCACAATGTCTCCTCC-3′ (forward) and 5′-GAGGAACTCTGGGAATGTGC-3′ (reverse); CTSL, 5′-GTGGACCAAGTGGAAGGC-3′ (forward) and 5′-CTCCAAAGGCGTTCATGG-3′ (reverse); APOD, 5′-CCTTTGAGAATGGACGCTGC-3′ (forward) and 5′-AGTTCTCATAGTCGGTGGCC-3′ (reverse); and C1QL1, 5′-ACGACGTGGTCACCAACC-3′ (forward) and 5′-TAGTTCTGGTCCGCGTCC-3′ (reverse). mRNA expression levels were normalized by the geometric mean of the housekeeping genes HPRT1, HMBS, and RPL13A, as described previously [34].

### 2.5. Western Blotting

Whole cell lysates were prepared using ice-cold RIPA buffer (50 mMTris-HCl pH 8.0, 150 mMNaCl, 0.1% Triton X-100, 0.1% SDS, 0.5% sodium deoxycholate) supplemented with Halt Protease Inhibitor Cocktail (Thermo Fisher Scientific). The protein concentration was determined using the Pierce BCA Protein Assay kit (Thermo Fisher Scientific). Equal amounts (20 μg) of protein were separated on a 12% Bis–TrisNuPage precast gel (Thermo Fisher Scientific) and transferred to a Hybond-C membrane (GE Healthcare Life Sciences, Marlborough, MA, USA) using the iBlot dry blotting system (Thermo Fisher Scientific). The membranes were immunoblotted with appropriate primary antibodies at 4 °C and subsequently with HRP-conjugated secondary antibody (ab6721, Abcam, Cambridge, UK) for 1 h atRT. The following primary antibodies were used: anti-APOD (RD181118100, BioVendor, Brno, Czech Republic), anti-C1QL1 (ab68528, Abcam), and anti-CTSL (GTX26314, GeneTex, Irvine, CA, USA). Specific bands were visualized using WESTAR ETA C 2.0 (XLS070,0250) (Cyanagen, Bologna, Italy) and the G-Box chemiluminescence imaging station (Syngene, Cambridge, UK).

### 2.6. Analysis of Interleukin Production

Cell culture supernatants were analyzed by enzyme-linked immunosorbent assay (ELISA) using the following kits according to the manufacturer’s protocols: kits for apolipoprotein D (APOD) (SEB968Hu) and complement component 1, Q subcomponent-like protein 1 (C1QL1) (SEE867Hu) from Cloud Clone (Wuhan, Hubei, PRC); Human IL-2 Quantikine ELISA, Human IL-4 Quantikine ELISA, and Human IL-17 Quantikine ELISA kits from Bio-Techne (Minneapolis, MN, USA); and a Human CTLA4 ELISA kit from Nordic Biosite (Täby, Sweden). Microtiter plates were read in a Sunrise microplate reader (Tecan).

### 2.7. Chromatin Immunoprecipitation Assay

A chromatin immunoprecipitation assay was performed using the EZ-Magna ChIP A/G Kit from EMD Millipore (Billerica, MA, USA). The following antibodies were used: normal mouse IgG (EMD Millipore), anti-H3K4me3 antibody (C15410030; Diagenode, Seraing, Belgium), anti-H3K27me2/3 antibody (Mab-014-050; Diagenode), and anti-H2BK12ac antibody (C15410212; Diagenode). To detect the immunoprecipitated promoter regions, quantitative PCR was performed on a LightCycler 96 system from Roche with the following primer pairs: APOD, 5′-GATTCTGCATCTGGAAACTGC-3′ (forward) and 5′-CTAAAGCTACCGGCACAAGC-3′ (reverse); CTSL, 5′- GTCTTTTCAGGAGCCACTCG-3′ (forward) and 5′- GACTCTGGGTGCTCTGATCC-3′ (reverse); and C1QL1, 5′-CCCAGTCCCTCTGTTTTCC-3′ (forward) and 5′-AGACTGAGTACGCGGAGACC-3′ (reverse). The PCR conditions were 95 °C for 10 min, followed by 45 cycles of 95 °C for 10 s, 60 °C for 20 s, and 72 °C for 20 s. Chromatin collected before immunoprecipitation was used as an input control. The relative PCR product enrichment was calculated using thedCtmethod with the Ct obtained for input DNA as a reference value as follows: 1000* 2-dCt, where dCt = Ct sample – Ct input DNA.

### 2.8. Statistics

Statistical analysis was performed using one-way ANOVA followed by Dunn’s post hoc test or Friedman repeated measures ANOVA on ranks followed by Dunn’s post hoc test. A P-value of 0.05 or lower indicated statistical significance. Statistical analyses were performed using SigmaStat version 3.5 (Systat Software, Inc., San Jose, CA, USA).

## 3. Results

### 3.1. Identification of Novel Th17-Specific Genes

**Figure 1:**
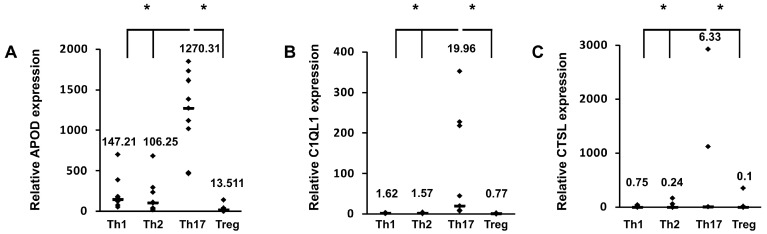
mRNA expression of the *APOD* (**A**), *C1QL1* (**B**), and *CTSL* (**C**) genes in Th1, Th2, Th17, and Treg cells. The expression of cognate genes was determined by real-time RT-PCR and normalized to that of the housekeeping genes *HPRT1*, *HMBS* and *RPL13A*. The data are presented as statistical dot plots with the median value (bars) from nine independent experiments performed using cultures from nine different donors (*n* = 9). An asterisk indicates a statistically significant difference at *p* < 0.05. Statistical analysis was performed using Friedman repeated measures ANOVA on ranks followed by Dunn’s post hoc test.

**Figure 2:**
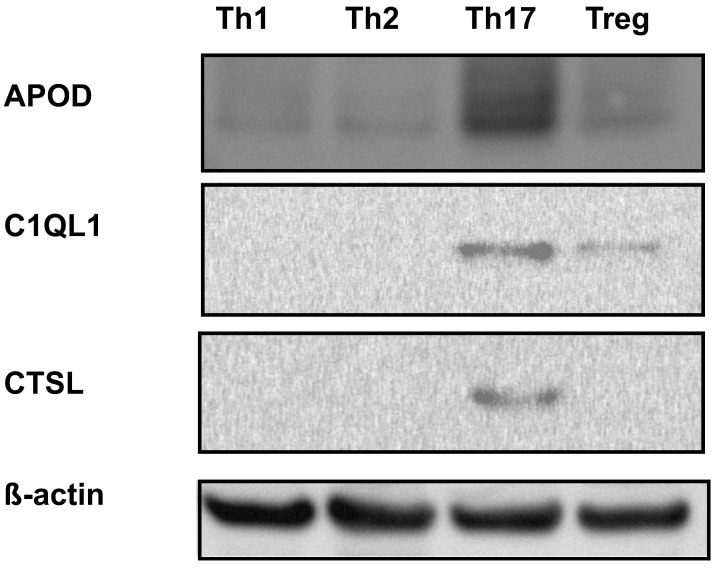
Representative Western blot detection of the APOD, C1QL1, and CTSL proteins in Th1, Th2, Th17 and Treg cells originating from CD4+ cells isolated from buffy coats. β-Actin was used as a loading control.

**Figure 3:**
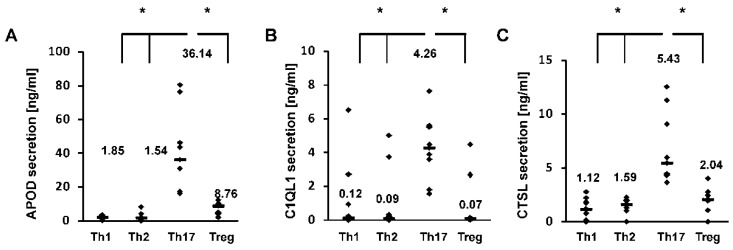
Results of APOD (**A**), C1QL1 (**B**), and CTSL (**C**) production determined using ELISA. The data are presented as statistical dot plots with the median value (bars) from nine independent experiments performed using cultures from nine different donors (*n* = 9). An asterisk indicates a statistically significant difference at *p* < 0.05. Statistical analysis was performed using Friedman repeated measures ANOVA on ranks followed by Dunn’s post hoc test.

To identify new Th17 cell markers, in the first stage of our study, we performed de novo sequencing of naive CD4+ cells and cells differentiated towards a Th17 phenotype for five days. Transcriptomics analysis revealed that the expression of more than 2000 genes differed in Th17 lymphocytes in comparison to CD4+ cells (Dataset S1). Some of these differentially expressed genes were previously identified, e.g., *RORC* [5], *VDR* [35], *BATF3* [36], *HIF1A* [37], *ATP1B1*, *IL2RB*, *COL6A3*, and *MIAT* [38] (Dataset S1). Ontological analysis revealed that many terms linked to the biology and differentiation of T cells were enriched in the differentially expressed genes (Table S1 and Dataset S2). In the next step, we selected several dozen genes, which we screened using real-time RT-PCR to detect their expression in cells isolated from two blood donors differentiated towards Th1 cells, Th2 cells, Th17 cells, and Tregs. Based on the results of this experiment, three putative Th17-specific genes were selected: *APOD*, *C1QL1*, and *CTSL* (Table S2). These genes were then analyzed in detail in cells isolated from a larger group of donors (Figure 1). The cell differentiation of each subpopulation was confirmed by expression of the signature transcription factors RORγT, TBX21, GATA3, and FOXP3 [6,39,40,41] and secretion of the specific cytokines/proteins IL-2, IL4, IL17, and CTLA4 [42,43,44,45,46] (Figures S1 and S2). We showed that at the mRNA level, the expression of these genes was significantly lower in different T cell subtypes than in Th17 cells, in which their expression was at least one log higher (Figure 1), analogous to the expression of RORγT (Figure S1C). Similar results were obtained using Western blotting (Figure 2). Because both APOD, C1QL1, and CTSL are secreted proteins, we determined their amounts in the cell supernatants. As expected, we observed the highest median APOD protein level in the supernatant of Th17 cells (36.14 ng/mL) in comparison to those of Th1 cells (1.85 ng/mL), Th2 cells (1.54 ng/mL), and Tregs (8.76 ng/mL) (Figure 3A). A similar pattern was observed for C1QL1, which exhibited median expression levels of 4.26 ng/mL (Th17 cells), 0.12 ng/mL (Th1 cells), 0.09 ng/mL (Th2 cells), and 0.07 ng/mL (Tregs) (Figure 3B), and for CTSL, with median expression levels of 5.43 ng/mL (Th17 cells), 1.12 ng/mL (Th1 cells), 1.59 ng/mL (Th2 cells), and 2.04 ng/mL (Tregs) (Figure 3C).

### 3.2. Analysis of Epigenetic Marks in the Loci of the Identified Genes

**Figure 4:**
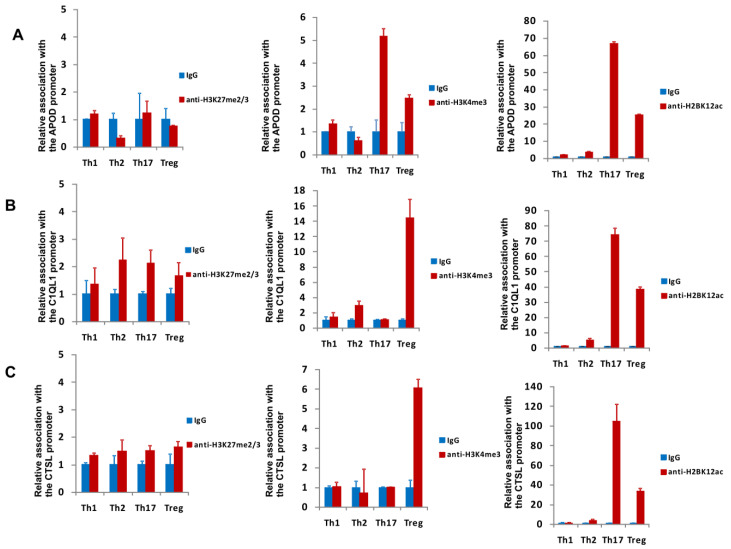
Analysis of the binding of H3K27me2/3, H3K4me3, and H2BK12ac in vivo to the promoter sequences of the *APOD* (**A**), *C1QL1* (**B**), and *CTSL* (**C**) genes in Th1, Th2, Th17, and Treg cells by chromatin immunoprecipitation. The results are shown as the mean ± SD, *n* = 3 (different donors).

As a next step, we examined the methylation and acetylation patterns of histones bound to the promoter regions of the identified genes in Th1 cells, Th2 cells, Th17 cells, and Tregs in vivo using chromatin immunoprecipitation (ChIP) assays to determine whether some cell-type-specific epigenetic modifications would be observed. The following modifications were analyzed: both H3K4me3 and H2BK12ac were associated with the activation status of the genes, and H3K27me2/3 was associated with inactive gene promoters [47,48]. Interestingly, the *CTSL1* and *C1QL1* gene promoters exhibited high levels of H3K4me3 (Figure 4) in Tregs, whereas for the *APOD* gene, the greatest degree of H3K4me3 binding was observed in Th17 cells, and that in Tregs was significantly lower (Figure 4A). Upon comparing the H2BK12ac occupancy of the *APOD*, *CTSL*, and *C1QL1* promoters in Th1, Th2, Th17, and Th17 cells, we observed that H2BK12ac levels were highest in Th17 cells, although it should be noted that the H2BK12ac occupancy in Tregs was also substantial (Figure 4A–C). *CTSL1* promoter-, *C1QL1* promoter-, and *APOD* promoter-bound H3K27me2/3 was not detectable or observed at very low amounts and did not show a T-cell-specific pattern (Figure 4A–C).

## 4. Discussion

In the present study, using transcriptional profiling of human naive CD4+ and Th17 cells, we selected genes potentially upregulated in mature Th17 cells. Then, we tested the expression of these genes in a number of cultures initiated from different donors to identify genes whose expression was potentially specific to Th17 cells (in comparison to Th1 cells, Th2 cells, and Tregs) (Table S2). Through this analysis, we identified three candidates, *APOD*, *C1QL1*, and *CTSL*, which were examined in a larger number of donors and whose specificity for Th17 cells was confirmed at the mRNA (Figure 1), cellular protein (Figure 2), and secreted protein levels (Figure 3). As already mentioned, among the identified genes was *APOD*, which encodes apolipoprotein D, a glycoprotein that is distinct from other apolipoprotein family members [49] with high similarity to the lipocalin family [50]. This protein transports a wide variety of molecules, including cholesterol, phospholipids, progesterone, and pregnenolone [50,51,52,53], as well as substances such as arachidonic acid [54] and (E)-3-methyl-2-hexenoic acid [55]. Previous proteomics analysis of human CD4+ and Th17 cells revealed that *APOD* is upregulated in Th17 cells [56], which is in line with our own observations (Dataset S1); however, our study went beyond this as it focused on the determination of T subset-restricted expression of this and other genes to identify novel markers of Th17 cells. Increases in *APOD* expression were found to be correlated with altered lipid metabolism and the risk of atherosclerosis [53]. Interestingly, patients with atherosclerosis also show significant increases in the number of peripheral Th17 cells and levels of Th17-related cytokines, e.g., IL-6, IL-17, and IL-23 [57]. Similar results were also confirmed in mouse models [58,59]. Thus, it is tempting to speculate that Th17 cell-derived APOD participates in the proinflammatory environment and might be a diagnostic factor in this and/or other Th17-dependent chronic inflammatory diseases, especially as patients with autoimmune diseases are at greater risk of developing arteriosclerosis [60,61]. Another gene identified in this study, *C1QL1*, encodes the secreted complement C1q-like 1 protein of unknown function, which is predominantly expressed in the brain [62,63] and exhibits affinity for the BAI3 receptor [64]. The protein product of this gene might act as a synaptic organizer [65], but the role of *C1QL1* outside the brain remains elusive. Liu et al. showed that *C1QL1* activates ERK1/2 and promotes angiogenesis [66]. It is generally accepted that inflammation fosters angiogenesis [67,68], and proinflammatory Th17 cells and their cytokines participate in this process [69,70,71,72]; thus, it is conceivable that *C1QL1* supports the Th17-dependent growth of new blood vessels under pathological conditions. *CTSL*, which encodes cathepsin L, a lysosomal cysteine proteinase, is another gene that revealed a Th17 cell-restricted expression pattern. Interestingly, Tuomela et al. identified it as among genes upregulated at the early stage of Th17 cell differentiation [38], but in striking contrast to our results, they also observed similar CTSL protein expression in Tregs. This discrepancy might be partially explained by the different cellular models used in the two studies. Tuomela et al. differentiated Tcells from the umbilical cord blood of neonates, while we used peripheral blood from adult donors. Neonates have a different immunological status compared to that of adults, which is related to the different gene expression patterns in immune cells [73,74]. Cathepsin L is involved in the regulation of CD4+ T cell selection in the thymus [75] and NKT cell development [76]. Recently, Hou et al. demonstrated that the endogenous cathepsin L inhibitor serpin B and other pharmacological inhibitors suppress Th17 differentiation, indicating that cathepsin L is an important player in promoting Th17 generation [77]. Furthermore, cathepsins including cathepsin L, when overexpressed, can be secreted and play a role in shaping the microenvironment in physiological and pathological processes, e.g., cancer [78] and various inflammatory disease including those with autoimmune components [79,80,81]. Our results suggest that this protein is specific for this particular Tcell subpopulation, in which it might be involved in the activation of receptors, cytokines, specific signaling proteins (e.g., STAT3) [82,83], and Th17-mediated tissue damage.

Because gene expression is determined by not only tissue-specific transcription factors but also by epigenetic mechanisms, we decided to determine whether we could find an epigenetic mark correlating with high expression of the identified genes in Th17 cells. Analysis of H3K27me2/3 binding, which is associated with gene silencing [47], within the promoters of the *APOD*, *C1QL1* and *CTSL* genes indicated that in all subtypes of Tcells, levels of this mark were low, or it was undetectable (Figure 4). Interestingly, when investigating two epigenetic marks that are enriched in the promoter regions of active genes, e.g., H3K4me3 and H2KB12ac [84,85,86], we found that H2BK12ac levels associated with the promoters of the *APOD*, *C1QL1* and *CTSL* genes were the highest in Th17 cells, which suggests a common epigenetic mechanism regulating Th17-restricted genes. However, unexpectedly, the H3K4me3 mark showed a different pattern (highest occupancy on the *C1QL1* and *CTSL* gene promoters in Tregs), and only the *APOD* gene was correlated with its expression in Th17 cells. Some epigenetic modifications may be dominant over others, e.g., H3K27me3 is usually dominant, which means that its presence is associated with repression [48,85]. H3K4me3 alone cannot induce active transcription [48,85], so it appears that the dominant modification in the case of the analyzed genes is H2KB12ac. It has been shown that H3K4me3 at the CNS2 locus of IL17A/F allows binding of the RORα and RORγT transcription factors and induction of the expression of IL17A/F during the differentiation of Th17 cells [87,88]. In response to some stimuli, Tregs can change their phenotype into a Th17-like phenotype [89,90,91]. It appears that epigenetic changes are involved in this process [92,93], and we suggest that H3K4me3 is a part of the histone code that maintains the region in an “open” state in the event that rapid activation associated with cellular plasticity is needed [94,95,96].

## 5. Conclusions

In summary, we present evidence that expression of the *APOD*, *C1QL1* and *CTSL* genes in human CD4+ cells is restricted to Th17 cells and associated with high levels of acetylated histone H2BK12 at the promoter regions of these genes. Furthermore, the expression of these genes and the functions of their encoded proteins might provide a better understanding of the involvement of Th17 cells in the pathogenic processes underlying arteriosclerosis and Th17 cell-driven angiogenesis. Furthermore, the results of analyses of the expression of these genes and concentrations of their protein products have potential clinical application in the identification of Th17 cell-related inflammation.

## Supplementary Materials

The following are available online at https://www.mdpi.com/2073-4409/9/7/1611/s1, Figure S1: mRNA expression of TBX21 (A), GATA3 (B),RORγT (C), and FOXP3 (D), signature transcription factor genes in Th1, Th2, Th17, and Treg cells, respectively. Figure S2: Results of IL-2 (A), IL-4 (B), IL-17 (C), and CTLA4 (D) production determined using ELISA. Table S1: Results of gene ontology (biological process) term analysis with the PANTHER overrepresentation test for significantly differentially expressed (DE) genes whose expression in CD4+ cells changed during differentiation towards a Th17 phenotype.Table S2: Results of initial screening for putative Th17-specific genes.DataSet S1. DataSet S2.
