# Supplementary material for: Identification of Novel Molecular Markers of Human Th17 Cells
Source: Cells. 2020 Jul 3;9(7):1611. doi: 10.3390/cells9071611 (PMC7407666; doi:10.3390/cells9071611)
Supplement: Supplementary file 1 [file cells-09-01611-s001.zip › Supplementary information.doc]

Supplementary information

Identification of novel molecular markers of human Th17 cells

Anna Sałkowska, Kaja Karaś, Iwona Karwaciak, Aurelia Walczak-Drzewiecka, Mariusz Krawczyk, Marta Sobalska-Kwapis, Jarosław Dastych and Marcin Ratajewski

Table of contents

1. Figure S1………………………………………………………………..S3
2. Figure S2………………………………………………………………..S4
3. Table S1…………………………………………………………………S5
4. Table S2…………………………………………………………………S6

Figure S1. mRNA expression of *TBX21* (A) *GATA3* (B) *RORγT* (C) and *FOXP3* (D), signature transcription factor genes in Th1, Th2, Th17 and Treg cells, respectively. The expression of cognate genes was determined by real-time RT-PCR and normalized to that of the housekeeping genes *HPRT1*, *HMBS* and *RPL13A*. The data are presented as statistical dot plots with the median value (bars) from nine independent experiments performed using cultures from nine different donors (n = 9). An asterisk indicates a statistically significant difference at P < 0.05. Statistical analysis was performed using Friedman repeated measures ANOVA on ranks followed by Dunn’s post hoc test.

Figure S2. Results of IL-2 (A), IL-4 (B), IL-17 (C), and CTLA4 (D) production determined using ELISA. The data are presented as statistical dot plots with the median value (bars) from nine independent experiments performed using cultures from nine different donors (n = 9). An asterisk indicates a statistically significant difference at P < 0.05. Statistical analysis was performed using Friedman repeated measures ANOVA on ranks followed by Dunn’s post hoc test.

Table S1. Results of gene ontology (biological process) term analysis with the PANTHER overrepresentation test for significantly differentially expressed (DE) genes whose expression in CD4+ cells changed during differentiation towards a Th17 phenotype. GOID: Gene Ontology term identifier; number of genes: the number of genes mapped to a specific GO term; p-value: the expected value calculated by Fisher’s exact test with false discovery rate multiple test correction (p< 0.05) for the overrepresentation of selected DE genes in the GO category.

| **GO ID** | **GO Biological Process Term** | **Number of Genes** | ***p*-Value** |
| --- | --- | --- | --- |
| GO:0002376 | immune system process | 299 | 2.47 × 10^-17^ |
| GO:0006955 | immune response | 220 | 3.86 × 10^-16^ |
| GO:0001819 | positive regulation of cytokine production | 81 | 5.42 × 10^-14^ |
| GO:0002682 | regulation of immune system process | 188 | 1.21 × 10^-13^ |
| GO:0001817 | regulation of cytokine production | 107 | 2.87 × 10^-13^ |
| GO:0030154 | cell differentiation | 354 | 5.13 × 10^-13^ |
| GO:0045321 | leukocyte activation | 118 | 4.22 × 10^-11^ |
| GO:0002443 | leukocyte mediated immunity | 100 | 6.12 × 10^-10^ |
| GO:0002694 | regulation of leukocyte activation | 82 | 5.64 × 10^-09^ |
| GO:0002685 | regulation of leukocyte migration | 38 | 1.14 × 10^-07^ |
| GO:0002521 | leukocyte differentiation | 52 | 1.82 × 10^-07^ |
| GO:0050863 | regulation of T cell activation | 44 | 2.24 × 10^-05^ |
| GO:0045580 | regulation of T cell differentiation | 25 | 5.89 × 10^-05^ |
| GO:0046649 | lymphocyte activation | 47 | 9.43 × 10^-05^ |
| GO:0030098 | lymphocyte differentiation | 32 | 4.58 × 10^-04^ |
| GO:2000514 | regulation of CD4-positive, alpha-beta T cell activation | 12 | 1.31 × 10^-03^ |
| GO:0070663 | regulation of leukocyte proliferation | 29 | 1.50 × 10^-03^ |

Table S2. Results of initial screening for putative Th17-specific genes. mRNA expression of the selected genes in Th1, Th2, Th17 and Treg cells was analyzed using real-time RT-PCR and normalized to that of the housekeeping genes *HPRT1*, *HMBS* and *RPL13A*.

| Gene | Donor 1 | | | | Donor 2 | | | |
| --- | --- | --- | --- | --- | --- | --- | --- | --- |
|  | Th1 | Th2 | Th17 | Treg | Th1 | Th2 | Th17 | Treg |
| *TNFRSF18* | 11.98 | 4.63 | 0.21 | 2.35 | 0.88 | 11.51 | 0.14 | 0.01 |
| *HIF1A* | 4 148.36 | 2 395.19 | 3 562.33 | 2 448.77 | 5 479.70 | 992.34 | 2 170.69 | 138.23 |
| *C1QL1* | 1.82 | 1.53 | 44.13 | 1.74 | 1.62 | 0.55 | 8.39 | 0.07 |
| *DHRS9* | 0.67 | 0.47 | 254.25 | 0.14 | 1.95 | 0.07 | 1.04 | 20.11 |
| *HMSD* | 115.80 | 24.35 | 473.94 | 25.77 | 144.65 | 58.39 | 144.77 | 1.05 |
| *MT3* | 97.68 | 38.85 | 204.25 | 10.75 | 116.61 | 3.74 | 43.94 | 0.23 |
| *EPAS1* | 276.35 | 953.25 | 663.60 | 159.53 | 244.16 | 683.93 | 46.00 | 7.20 |
| *IL1R2* | 39.10 | 65.00 | 548.22 | 562.22 | 100.74 | 24.97 | 72.68 | 32.81 |
| *TMPRSS6* | 3.63 | 5.05 | 11.91 | 16.20 | 2.00 | 2.76 | 7.12 | 0.74 |
| *CD22* | 4.13 | 1.52 | 40.80 | 362.70 | 8.91 | 7.50 | 2.94 | 10.86 |
| *DUSP6* | 168.37 | 771.81 | 300.47 | 55.10 | 282.32 | 298.64 | 49.92 | 3.29 |
| *RASGRP4* | 0.28 | 0.90 | 0.96 | 5.60 | 0.22 | 0.29 | 0.95 | 0.25 |
| *CTSL1* | 23.36 | 165.03 | 1 122.54 | 351.57 | 43.27 | 59.15 | 2 922.45 | 13.84 |
| *CD8A* | 4.64 | 10.38 | 0.06 | 6 480 446 687.59 | 1.69 | 1.53 | 2.14 | 1 276 936 357.28 |
| *PTPN14* | 8.42 | 162.39 | 154.85 | 6.29 | 55.70 | 50.81 | 75.20 | 0.21 |
| *ALPK2* | 0.78 | 0.16 | 0.88 | 0.32 | 37.02 | 0.30 | 31.98 | 0.03 |
| *CD109* | 28.25 | 67.45 | 118.08 | 243.33 | 219.77 | 27.74 | 118.01 | 13.46 |
| *APOD* | 74.64 | 234.72 | 1 270.31 | 137.38 | 123.12 | 106.25 | 1 613.69 | 15.94 |
| *LGALS14* | 10.20 | 3.60 | 1.49 | n/a | 10.68 | 3.39 | 0.65 | n/a |
| *NIPAL4* |  |  |  |  |  |  |  |  |
| *TNP1* | 0.01 | 0.07 | 0.03 | 0.41 | 0.02 | 0.08 | 0.06 | 0.02 |
| *CFAP57* |  |  |  |  |  |  |  |  |
| *MYO18B* | 157.26 | 114.90 | 209.93 | 934.88 | 258.08 | 191.02 | 205.71 | 446.49 |
| *C1orf106* | 0.03 | 0.06 | 0.01 | n/a | 0.06 | 0.04 | 0.07 | n/a |
| *SYP* | 25.91 | 42.20 | 12.64 | 4.98 | 20.59 | 31.70 | 13.89 | 9.12 |
| *NAPSB* | 71.73 | 29.02 | 705.11 | 0.45 | 150.03 | 4.72 | 34.23 | 47.51 |
| *SCD* | 1 259.66 | 1 472.35 | 1 399.37 | 1 645.50 | 1 339.98 | 1 145.95 | 728.39 | 108.05 |
| *VWCE* | 3.51 | 3.90 | 4.77 | 6.92 | 9.98 | 31.72 | 6.95 | 0.95 |
| *REEP2* | 10.01 | 12.19 | 40.25 | 46.33 | 10.21 | 13.24 | 46.92 | 5.08 |
| *CDH1* | 18.24 | 2.48 | 49.90 | 36.05 | 116.28 | 27.00 | 106.66 | 86.86 |
| *IL2RA* | 8 602.27 | 4 539.27 | 5 371.06 | 4 384.75 | 3 983.14 | 2 534.01 | 727.96 | 1 020.83 |
| *SEMA7A* | 221.33 | 107.50 | 207.81 | 50.88 | 188.59 | 90.94 | 213.32 | 124.26 |
| *DDIT4* | 301.05 | 179.05 | 423.37 | 136.61 | 216.74 | 68.69 | 96.88 | 61.42 |
| *CD38* | 1 881.46 | 2 689.91 | 105.11 | 379.75 | 1 947.52 | 232.64 | 219.50 | 86.83 |

Original blot scans.

APOD

From L-R protein ladder, Th1, Th2, Th17, Treg

CTSL

From L-R protein ladder, Th1, Th2, Th17, Treg, protein ladder

C1QL1

From L-R protein ladder, Th1, Th2, Th17, Treg

β-actin

From L-R Th1, Th2, Th17, Treg
